# Supplementary material for: Prevalence characteristics of cervical human papillomavirus infection in Chengdu and Aba District, Sichuan Province, China
Source: PLoS One. 2024 Jun 13;19(6):e0304760. doi: 10.1371/journal.pone.0304760 (PMC11175428; doi:10.1371/journal.pone.0304760)
Supplement: S1 File — (DOCX) [file pone.0304760.s001.docx]

**Table S1-A Genotype-specific distribution of HPV infections in Chengdu.**

| **Genotypes** | **Single infection**  **No. (%)** | **Multiple infections**  **No. (%)** | **Total***  **No. (%)** |
| --- | --- | --- | --- |
| 52 | 4399 (3.05) | 2859 (1.98) | 7258 (5.04) |
| 16 | 2540 (1.76) | 1951 (1.35) | 4491 (3.12) |
| 58 | 2167 (1.50) | 1617 (1.12) | 3784 (2.63) |
| 81 | 1863 (1.29) | 1686 (1.17) | 3549 (2.46) |
| 53 | 1766 (1.23) | 1524 (1.06) | 3290 (2.28) |
| 51 | 1542 (1.07) | 1371 (0.95) | 2913 (2.02) |
| 68 | 1045 (0.73) | 893 (0.62) | 1938 (1.34) |
| 56 | 915 (0.63) | 907 (0.63) | 1822 (1.26) |
| 59 | 865 (0.60) | 957 (0.66) | 1822 (1.26) |
| 42 | 788 (0.55) | 942 (0.65) | 1730(1.20) |
| 18 | 904 (0.63) | 800 (0.56) | 1704 (1.18) |
| 43 | 813 (0.56) | 844 (0.59) | 1657 (1.15) |
| 33 | 664 (0.46) | 712 (0.49) | 1376 (0.95) |
| 66 | 615 (0.43) | 717 (0.50) | 1332 (0.92) |
| 6 | 591 (0.41) | 738 (0.51) | 1329 (0.92) |
| 39 | 634 (0.44) | 642 (0.45) | 1276 (0.89) |
| 31 | 430 (0.30) | 423 (0.29) | 853 (0.59) |
| 11 | 439 (0.30) | 383 (0.27) | 822 (0.57) |
| 35 | 292 (0.20) | 384 (0.27) | 676 (0.47) |
| 45 | 223 (0.15) | 229 (0.16) | 452 (0.31) |
| 73 | 95 (0.07) | 135 (0.09) | 230 (0.16) |
| 82 | 107 (0.07) | 107 (0.07) | 214 (0.15) |
| 83 | 79 (0.05) | 115 (0.08) | 194 (0.13) |
| Total | 23783 (16.50) | 8660 (6.01) | 32443 (22.51) |

^*^ P< 0.001. This table contains values used to build Fig1A.

**Table S1-B Genotype-specific distribution of HPV infections in Aba.**

| **Genotypes** | **Single infection**  **No. (%)** | **Multiple infections**  **No. (%)** | **Total***  **No. (%)** |  |
| --- | --- | --- | --- | --- |
| 52 | 42 (2.33) | 20 (1.11) | 62 (3.45) |  |
| 53 | 19 (1.06) | 18 (1.00) | 37 (2.06) |  |
| 16 | 26 (1.45) | 8 (0.44) | 33 (1.83) |  |
| 18 | 15 (0.83) | 8 (0.44) | 23 (1.28) |  |
| CP8304 | | 12 (0.67) | 11 (0.61) | 23 (1.28) |
| 58 | 12 (0.67) | 10 (0.56) | 22 (1.22) |  |
| 31 | 18 (1.00) | 3 (0.17) | 21 (1.17) |  |
| 56 | 10 (0.56) | 8 (0.44) | 18 (1.00) |  |
| 51 | 8 (0.44) | 10 (0.56) | 18 (1.00) |  |
| 39 | 8 (0.44) | 9 (0.50) | 17 (0.94) |  |
| 45 | 8 (0.44) | 4 (0.22) | 12 (0.67) |  |
| 33 | 4 (0.22) | 5 (0.28) | 9 (0.50) |  |
| 66 | 2(0.11) | 7 (0.39) | 9 (0.50) |  |
| 68 | 2 (0.11) | 4 (0.22) | 6 (0.33) |  |
| 35 | 4 (0.22) | 2 (0.11) | 6 (0.33) |  |
| 43 | 3 (0.17) | 3 (0.17) | 6 (0.33) |  |
| 6 | | 3 (0.17) | 2 (0.11) | 5 (0.28) |
| 59 | | 0 | 1 (0.06） | 1 (0.06) |
| 11 | 0 | 1 (0.06) | 1 (0.06) |  |
| Total | 196 (10.89) | 60 (3.34) | 256 (14.23) |  |

^*^ P< 0.001. This table contains values used to build Fig1B.

**Table S2-A Prevalence of HPV infections in Chengdu from 2015 to 2020(%):**

**Prevalence of HPV genotypes in single, multiple and total infections.**

| **Year** | **Single infection**  **No. (%)** | **Multiple infections**  **No. (%)** | **Total***  **No. (%)** |  |
| --- | --- | --- | --- | --- |
| 2015 | 3184 (15.61) | 1284 (6.30) | 4468 (21.91) |  |
| 2016 | 3192 (15.36) | 1146 (5.51) | 4338 (20.87) |  |
| 2017 | 4214 (17.61) | 1426 (5.96) | 5640 (23.56) |  |
| 2018 | 5472 (18.32) | 1986 (6.65) | 7458 (24.98) |  |
| 2019 | | 5187 (15.69) | 1846 (5.58) | 7033 (21.28) |
| 2020 | 2535 (15.77) | 971 (6.04) | 3506 (21.81) |  |
| Total | 23784 (16.50) | 8659 (6.01) | 32443 (22.51) |  |
| P | <0.001 | <0.001 | <0.001 |  |

This table contains values used to build Fig 2A.

**Table S2-B Prevalence of HPV infections in Chengdu from 2015 to 2020(%): Changes in the prevalence of the top six HPV genotypes.**

|  | |  | **Genotypes No. (%)** | | | |
| --- | --- | --- | --- | --- | --- | --- |
| **Year** | **52** | **16** | **58** | **81** | **53** | **51** |
| 2015 | 1059 (5.19) | 593 (2.91) | 516 (2.53) | 581 (2.85) | 470 (2.30) | 409 (2.01) |
| 2016 | 970 (4.67) | 605 (2.91) | 441 (2.12) | 438  (2.11) | 407 (1.96) | 425 (2.04) |
| 2017 | 1228 (5.13) | 732 (3.06) | 598 (2.50) | 554 (2.31) | 581 (2.43) | 508 (2.12) |
| 2018 | 1553 (5.20) | 1075 (3.60) | 854 (2.86) | 743 (2.49) | 731 (2.45) | 687 (2.30) |
| 2019 | 1612 (4.88) | 996 (3.01) | 876 (2.65) | 832 (2.52) | 706 (2.14) | 590 (1.78) |
| 2020 | 836 (5.20) | 490 (3.05) | 499 (3.10) | 401 (2.49) | 395 (2.46) | 294 (1.83) |
| P | <0.05 | <0.001 | <0.001 | <0.001 | <0.05 | <0.001 |

This table contains values used to build Fig 2B.

**Table S3-A Prevalence of HPV infections in Aba from 2018 to 2021(%):**

**Prevalence of HPV genotypes in single, multiple and total infections.**

| **Year** | **Single infection**  **No. (%)** | **Multiple infections**  **No. (%)** | **Total***  **No. (%)** |
| --- | --- | --- | --- |
| 2018 | 5 (6.25) | 2 (2.50) | 7 (8.75) |
| 2019 | 37 (7.13) | 14 (2.70) | 51 (9.83) |
| 2020 | 30 (10.00) | 7 (2.33) | 37 (12.33) |
| 2021 | 124 (13.78) | 37 (4.11) | 161 (17.89) |
| Total | 196 (10.89) | 60 (3.34) | 256 (14.23) |
| P | <0.05 | 0.328 | <0.001 |

This table contains values used to build Fig 3A.

**Table S3-B Prevalence of HPV infections in Aba from 2018 to 2021(%): Changes in the prevalence of the top six HPV genotypes.**

|  | |  | **Genotypes No. (%)** | | | |
| --- | --- | --- | --- | --- | --- | --- |
| **Year** | **52** | **53** | **16** | **18** | **CP8304** | **58** |
| 2018 | 0 (0) | 1 (1.25) | 2 (2.50) | 0 (0) | 1 (1.25) | 1 (1.25) |
| 2019 | 10 (1.93) | 7 (1.35) | 6 (1.16) | 3 (0.58) | 2 (0.39) | 5 (0.96) |
| 2020 | 9 (3.00) | 5 (1.67) | 3 (1.00) | 2 (0.67) | 6 (2.00) | 8 (2.67) |
| 2021 | 25 (2.78) | 17 (1.89) | 23 (2.56) | 18 (2.00) | 14 (1.56) | 8 (0.89) |
| P | 0.335 | 0.879 | 0.165 | 0.054 | 0.167 | 0.098 |

This table contains values used to build Fig 3B.

**Table S4-A The prevalence of six most prevalent HPV types by age groups:** **the epidemic characteristics in Chengdu.**

|  | |  | **Genotypes No. (%)** | | | |
| --- | --- | --- | --- | --- | --- | --- |
| **Age(years)** | **52** | **16** | **58** | **81** | **53** | **51** |
| ≤25 | 1197 (6.45) | 817 (4.40) | 632 (3.40) | 561 (3.02) | 525 (2.83) | 591 (3.18) |
| 26-35 | 3686 (4.75) | 2153 (2.77) | 1781 (2.29) | 1607 (2.07) | 1546 (1.99) | 1481 (1.91) |
| 36-45 | 1309 (4.56) | 795 (2.77) | 727 (2.53) | 742 (2.58) | 642 (2.24) | 481 (1.67) |
| ≥46 | 1066 (5.55) | 726 (3.78) | 644 (3.35) | 639 (3.33) | 577 (3.01) | 360 (1.88) |
| P | <0.001 | <0.001 | <0.001 | <0.001 | <0.001 | <0.001 |

This table contains values used to build Fig 4A.

**Table S4-B The prevalence of six most prevalent HPV types by age groups:** **the epidemic characteristics in Aba.**

|  | |  | **Genotypes No. (%)** | | | |
| --- | --- | --- | --- | --- | --- | --- |
| **Year** | **52** | **53** | **16** | **18** | **CP8304** | **58** |
| ≤25 | 4 (6.90) | 1 (1.72) | 1 (1.72) | 2 (3.45) | 2 (3.45) | 0 (0) |
| 26-35 | 10 (2.88) | 10 (2.88) | 10 (2.88) | 3 (0.86) | 3 (0.86) | 5 (1.44) |
| 36-45 | 20 (3.25) | 11 (1.79) | 10 (1.63) | 5 (0.81) | 7 (1.14) | 7 (1.14) |
| ≥46 | 28 (3.59） | 15 (1.93） | 13 (1.67) | 13 (1.67） | 11 (1.47) | 10 (1.28) |
| P | 0.470 | 0.684 | 0.515 | 0.201 | 0.417 | 0.822 |

This table contains values used to build Fig 4B.
