# Supplementary material for: Prevalence characteristics of cervical human papillomavirus infection in Chengdu and Aba District, Sichuan Province, China
Source: PLoS One. 2024 Jun 13;19(6):e0304760. doi: 10.1371/journal.pone.0304760 (PMC11175428; doi:10.1371/journal.pone.0304760)
Supplement: Attachment — Submitted filename: Response to Reviewers.docx [file pone.0304760.s002.docx]

Dear Editors and Reviewers:

Thank you for your letter and for the reviewers' comments concerning our manuscript entitled “Prevalence characteristics of cervical human papillomavirus infection in Chengdu and Aba District, Sichuan Province, China” (ID: PONE-D-23-41668). Those comments are all valuable and very helpful for revising and improving our paper, as well as the important guiding significance to our research. We have studied comments carefully and have made corrections which we hope meet with approval. Revised portions are marked in red on the paper. The main corrections in the paper and the responses to the reviewer's comments are as follows:

Responds to the editor's and reviewer's comments:

**Editor:**

Response to comment: However, thinking a novelty, the authors should put more emphasis on their novel findings based on the database and claim some suggestions on how to reduce the burden of cervical cancer in China.

Response: We have added some suggestions in the conclusion part of the discussion (line 360-367 in the file labeled “Revised Manuscript with Track Changes”) as follows: Based on the age profile of HPV prevalence in the two districts, it is recommended that the age range for free screening be expanded. Our data also showed a high prevalence of some non-vaccine genes, making the development of region-specific vaccines imminent. HPV vaccination programs can protect from HPV infection and then reduce the future burden of invasive cervical cancer, but the current cervical cancer vaccine is in short supply and expensive to administer, so it is hoped that the cost of the vaccine will be covered by insurance or health insurance in the future.

**Reviewer #1:**

Response to comment: Suggested to discuss and compare with the distribution of any of these study prevalent types with the regions other than China and other continents also.

Response: We have added comparisons with other regions outside of China (line 255-260 and line 281-284 in the file labeled “Revised Manuscript with Track Changes”) as follows:

A meta-analysis of HPV prevalence in cytologically normal women from five continents showed that the global HPV prevalence was 11.7%. The continents, in descending order of prevalence, were: Africa (21.1%), Europe (14.2%), America (11.5%), and Asia (9.4%). Eastern Asia (10.7%) had the second highest infection rate in Asia following Southeastern Asia (14.0%).

According to a report, HPV-16, HPV-18, HPV-52, HPV-31, and HPV-58 were the five most prevalent forms globally. In contrast, HPV-16, HPV-18, HPV-52, HPV-51, and HPV-58 were the most prevalent types in Asia.

**Reviewer #2:**

Response to comment: The data provided in this study may be helpful for government-level decision-making, but revision is required.

Response: Special thanks to you for your good comments, and we will modify them one by one as required.

**Major comments:**

1. Response to comment: The two units mentioned in this study are administratively closely related (one is a subordinate of the other one)? If not, the ethical approval from the second unit is also required.

Response: The two units mentioned in this study are not administratively closely related, but the Aba Maternal and Child Health Hospital is a supported unit of the Chengdu Women's and Children's Center Hospital. Since Aba Maternal and Child Health Hospital is a primary care unit without a specialized ethics committee, we were unable to provide the appropriate ethical approvals, but we followed the Declaration of Helsinki, including confidentiality and anonymity, throughout the study.

2. Response to comment: The population included in the two units are possibly different. Participants from the Chengdu hospital tended to be patients (Outpatients) and residents (physical examination) while those from the Aba hospital tended to be from a cancer screening programme (possibly dominated by patients or residents, but not both, and most probably the latter) which could have contributed to the significant difference between the two regions. The authors didn’t mention this point in the discussion section.

Response: It is true as the Reviewer mentioned the samples from the two units may be different, which may contribute to the significant difference between the two. Therefore, we have removed the relevant comparisons between the two regions through the article and present a parallel presentation of the results of the two regions. The specific changes in the file labeled “Revised Manuscript with Track Changes” are as follows:

Line 194-195: “Between the two areas, significant differences in HPV prevalence were observed (P < 0.001), and details of genotype distribution are presented below” was deleted.

Line 253-255: “Regarding the overall HPV prevalence in the two distinct regional groups, this survey has revealed significant differences: Chengdu (22.51%) and Aba (14.23%)” was corrected as “The overall HPV prevalence in the two distinct regional groups showed 22.51% in Chengdu and 14.23% in Aba”.

Line 269-271: “The overall prevalence in Chengdu was found to be higher than that in Aba. This could potentially be attributed to the lower screening rates observed in Aba, as evidenced by its lesser annual prevalence” was deleted.

Line 284: “Our study found variations between two regions” were corrected as “Our study found that”.

Line 355-358: “To summarize, our study has estimated the prevalence of HPV infection among females in Chengdu and Aba of Sichuan Province and there are notable differences between the two areas in HPV infection rates, annual trends, age-specific prevalence, and type distribution.” was corrected as “To summarize, our study has estimated the prevalence of HPV infection rates, annual trends, age-specific prevalence, and type distribution in Chengdu and Aba of Sichuan Province”.

3. Response to comment: There are claims not supported by the data provided in the manuscript, such as in line 314-317, the authors concluded the difference in the awareness of preventive measures, but in the manuscript data supporting such claim is not to be found.

Response: As for the Reviewer’s suggestion, we have deleted the “awareness of HPV prevention” in line 251-252 and line 337-340 in the file labeled “Revised Manuscript with Track Changes”, given that this paper is not supported by direct data.

4. Response to comment: The genotyping kits used in the study differ in the number of total HPV subtypes covered, which should have contributed to the observed difference in the prevalence, however, the authors didn’t mention its impact to the final results.

Response: Given that the genotyping kits used in the two regions in this study did differ in terms of the total HPV subtypes covered, it would indeed be uncritical to make a direct comparison between the two regions. Therefore, we have eliminated the comparisons between the two regions and directly presented the results of the two ones.

5. Response to comment: Is CP8304 genotype 81? Check this out and make corrections.

Response: HPV81 and HPV CP8304 belong to different genotypes.

**Minor points:**

Response to comment: the manuscript requires extensive revision to correct for incorrect use of words and inappropriate sentences. To list just a few: line 62-64; line 65-66; line 72-73; line 76-77; line 88; line 108; line 284-286......

Response:

1. Line 62-64 are equivalent to line 66-68 in the file labeled “Revised Manuscript with Track Changes”: “In 2020, an estimated 604,000 new cases and 342,000 deaths were attributed to cervical cancer as the fourth most frequently diagnosed cancer and the fourth leading cause of cancer death in women” was corrected as “In 2020, an estimated 604,000 new cases and 342,000 deaths were attributed to cervical cancer which was the fourth most frequently diagnosed cancer and the fourth leading cause of cancer death in women”.
2. Line 65-66 are equivalent to line 69-71 in the file labeled “Revised Manuscript with Track Changes”: “Moreover, the majority of deaths occurred in developing countries rather than developed areas” was corrected as “Furthermore, developing countries saw a higher death rate than developed ones”.
3. Line 72-73 are equivalent to line 76-81 in the file labeled “Revised Manuscript with Track Changes”: “Unlike most malignant tumors, cervical cancer is considered nearly completely preventable benefit by the highly effective primary (vaccination) and secondary (screening) prevention measures” was corrected as “In contrast to most malignant tumors, cervical cancer is thought to be nearly completely preventable, benefiting from the highly effective primary (vaccination) and secondary (screening) preventative strategies”.
4. Line 76-77 are equivalent to line 83-85 in the file labeled “Revised Manuscript with Track Changes”: “High-quality screening strategies, including visual inspection with acetic acid, cervical cytology and HPV testing, are critical for unvaccinated women and for those infected with non-vaccine HPV types.” was corrected as “High-quality screening strategies, including visual inspection with acetic acid, cervical cytology, and HPV testing, are critical for unvaccinated women and those infected with non-vaccine HPV types”.
5. Line 88 is equivalent to line 95-96 in the file labeled “Revised Manuscript with Track Changes”: “Especially, there is a dearth of data from remote high-altitude localities” was corrected as “In particular, there is a lack of data from remote high-altitude locations”.
6. Line 108 is equivalent to line 117 in the file labeled “Revised Manuscript with Track Changes”: “Central Hospital since January 2015 to September 2020. Patients from outpatient” was corrected as “Central Hospital from January 2015 to September 2020. Patients from the outpatient”.
7. Line 284-286 are equivalent to line 302-306 in the file labeled “Revised Manuscript with Track Changes”: We apologize for not finding the point of error in the following sentence: “Furthermore, our analysis showed that HPV81 was the most prevalent low-risk HPV type in Chengdu, with the same tremendous advantage in other regions of China” was corrected as “Furthermore, our investigation revealed that HPV81, which has the same remarkable advantage in other parts of China, was the most common low-risk HPV type in Chengdu.”

We tried our best to improve the manuscript and made some changes in the manuscript. These changes will not influence the content and framework of the paper. Here we did not list some other changes but marked them in red in the revised paper.

We appreciate for Editors'/Reviewers' warm work earnestly and hope that the correction will meet with approval. Once again, thank you very much for your comments and suggestions.

Sincerely,

Qianqian Wang

Email: wangqianqian0330@126.com
