# Supplementary material for: Prevalence characteristics of cervical human papillomavirus infection in Chengdu and Aba District, Sichuan Province, China
Source: PLoS One. 2024 Jun 13;19(6):e0304760. doi: 10.1371/journal.pone.0304760 (PMC11175428; doi:10.1371/journal.pone.0304760)
Supplement: Attachment — Submitted filename: Response to Reviewers.docx [file pone.0304760.s003.docx]

Dear Editors and Reviewers:

Thank you very much for your attention and the reviewers' evaluation and comments on our paper entitled “Prevalence characteristics of cervical human papillomavirus infection in Chengdu and Aba District, Sichuan Province, China” (ID: PONE-D-23-41668R1). We deeply appreciate the reviewers' suggestion. According to the reviewers' comments, we have made corrections which we hope meet with approval. Revised portions are marked in red on the paper. The main corrections in the paper and the responses to the reviewer's comments are as follows:

**Reviewer #2:**

1. Response to comment: In the Abstract, the final sentence can be changed to '....HPV screening provides valuable guidance......'。

Response: In the Abstract, the final sentence was corrected as “In summary, regional HPV screening provides valuable clinical guidance for cervical cancer prevention and vaccine selection in Western China.”

2. Response to comment: Suggest change 'Furthermore, our investigation revealed that HPV81........other parts of China......' to 'Furthermore, our investigation revealed that HPV81, which exhibits similar prevalence as reported in other parts of China, was the most common low-risk HPV type in Chengdu'.

Response: We have changed “Furthermore, our investigation revealed that HPV81........other parts of China......” (in line 287-290 in the file labeled “Revised Manuscript with Track Changes”) to “Furthermore, our investigation revealed that HPV81, which exhibits similar prevalence as reported in other parts of China, was the most common low-risk HPV type in Chengdu”.

Thank you very much for all your help!

Sincerely,

Qianqian Wang

Email: wangqianqian0330@126.com
